# Dibenzyl­dichloridotin(IV)

**DOI:** 10.1107/S160053680901513X

**Published:** 2009-04-30

**Authors:** Kong Mun Lo, Seik Weng Ng

**Affiliations:** a Department of Chemistry, University of Malaya, 50603 Kuala Lumpur, Malaysia

## Abstract

The title compound, [Sn(C_7_H_7_)_2_Cl_2_], exists as a monomeric tetra­hedral mol­ecule. The Sn atom lies on a special position of site symmetry 2. Adjacent mol­ecules are linked into a linear chain running along the *b* axis of the monoclinic unit cell by Sn⋯Cl bridges of 3.7275 (4) Å.

## Related literature

For the synthesis of dibenzyl­tin dichloride by the direct reaction of benzyl chloride and metallic tin, see: Shishido *et al.* (1961 ▶). For an overview of crystallographic and theoretical structures of diorganotin dichlorides, see: Buntine *et al.* (2003 ▶). 
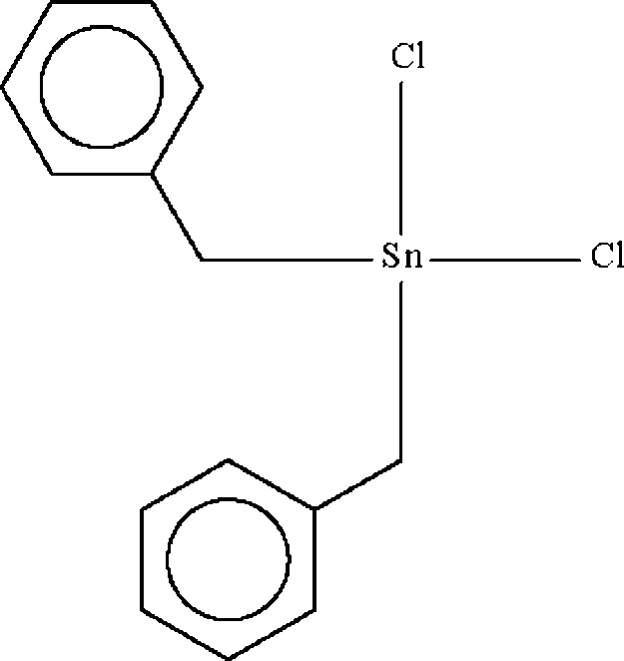


## Experimental

### 

#### Crystal data

[Sn(C_7_H_7_)_2_Cl_2_]
*M* *_r_* = 371.84
Monoclinic, 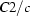

*a* = 23.7710 (3) Å
*b* = 4.8019 (1) Å
*c* = 12.0808 (2) Å
β = 92.560 (1)°
*V* = 1377.60 (4) Å^3^
*Z* = 4
Mo *K*α radiation
μ = 2.22 mm^−1^
*T* = 123 K
0.35 × 0.30 × 0.15 mm

#### Data collection

Bruker SMART APEX diffractometer
Absorption correction: multi-scan (*SADABS*; Sheldrick, 1996 ▶) *T*_min_ = 0.511, *T*_max_ = 0.732
6090 measured reflections
1580 independent reflections
1527 reflections with *I* > 2σ(*I*)
*R*_int_ = 0.021

#### Refinement

*R*[*F* ^2^ > 2σ(*F* ^2^)] = 0.014
*wR*(*F* ^2^) = 0.041
*S* = 1.03
1580 reflections
78 parameters
H-atom parameters constrained
Δρ_max_ = 0.27 e Å^−3^
Δρ_min_ = −0.60 e Å^−3^

### 

Data collection: *APEX2* (Bruker, 2008 ▶); cell refinement: *SAINT* (Bruker, 2008 ▶); data reduction: *SAINT*; program(s) used to solve structure: *SHELXS97* (Sheldrick, 2008 ▶); program(s) used to refine structure: *SHELXL97* (Sheldrick, 2008 ▶); molecular graphics: *X-SEED* (Barbour, 2001 ▶); software used to prepare material for publication: *publCIF* (Westrip, 2009 ▶).

## Supplementary Material

Crystal structure: contains datablocks global, I. DOI: 10.1107/S160053680901513X/tk2438sup1.cif

Structure factors: contains datablocks I. DOI: 10.1107/S160053680901513X/tk2438Isup2.hkl

Additional supplementary materials:  crystallographic information; 3D view; checkCIF report

## Figures and Tables

**Fig. 1.**
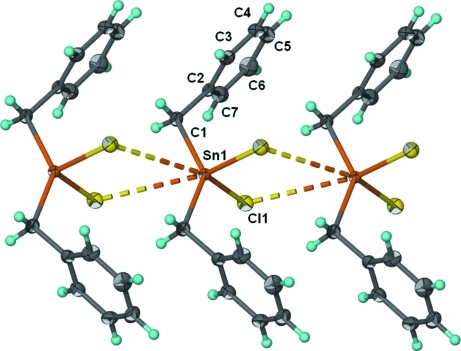
Thermal ellipsoid plot (Barbour, 2001) of part of the supramolecular chain in (C6H5CH2)2SnCl2 drawn at the 70% probability level. Dashed lines denote the Sn···Cl bridges. Hydrogen atoms are drawn as spheres of arbitrary radius. Unlabelled atoms within the partially labelled molecule are related by the symmetry operation: -x+1, y, -z+1/2.

## supplementary crystallographic information

### Experimental

Dibenzyltin dichloride was synthesized from benzyl chloride and metallic tin by a literature method (Shishido *et al.*, 1961). Crystals were obtained by recrystallization from chloroform.

### Refinement

Carbon-bound H-atoms were placed in calculated positions (C–H 0.95–0.99 Å) and were included in the refinement in the riding model approximation with *U*(H) set to 1.2*U*(C).

### Crystal data

**Table:**

[Sn(C_7_H_7_)_2_Cl_2_]	*F*(000) = 728
*M**_r_* = 371.84	*D*_x_ = 1.793 Mg m^−^^3^
Monoclinic, *C*2/*c*	Mo *K*α radiation, λ = 0.71073 Å
Hall symbol: -C 2yc	Cell parameters from 5461 reflections
*a* = 23.7710 (3) Å	θ = 2.5–28.3°
*b* = 4.8019 (1) Å	µ = 2.22 mm^−^^1^
*c* = 12.0808 (2) Å	*T* = 123 K
β = 92.560 (1)°	Block, colorless
*V* = 1377.60 (4) Å^3^	0.35 × 0.30 × 0.15 mm
*Z* = 4	

### Data collection

**Table:**

Bruker SMART APEX diffractometer	1580 independent reflections
Radiation source: fine-focus sealed tube	1527 reflections with *I* > 2σ(*I*)
graphite	*R*_int_ = 0.021
ω scans	θ_max_ = 27.5°, θ_min_ = 1.7°
Absorption correction: multi-scan (*SADABS*; Sheldrick, 1996)	*h* = −30→30
*T*_min_ = 0.511, *T*_max_ = 0.732	*k* = −6→6
6090 measured reflections	*l* = −15→15

### Refinement

**Table:**

Refinement on *F*^2^	Primary atom site location: structure-invariant direct methods
Least-squares matrix: full	Secondary atom site location: difference Fourier map
*R*[*F*^2^ > 2σ(*F*^2^)] = 0.014	Hydrogen site location: inferred from neighbouring sites
*wR*(*F*^2^) = 0.041	H-atom parameters constrained
*S* = 1.03	*w* = 1/[σ^2^(*F*_o_^2^) + (0.0238*P*)^2^ + 1.6061*P*] where *P* = (*F*_o_^2^ + 2*F*_c_^2^)/3
1580 reflections	(Δ/σ)_max_ = 0.001
78 parameters	Δρ_max_ = 0.27 e Å^−^^3^
0 restraints	Δρ_min_ = −0.60 e Å^−^^3^

### Fractional atomic coordinates and isotropic or equivalent isotropic displacement parameters (Å^2^)

**Table:**

	*x*	*y*	*z*	*U*_iso_*/*U*_eq_	
Sn1	0.5000	0.49246 (2)	0.2500	0.01354 (6)	
Cl1	0.473226 (16)	0.81293 (8)	0.38720 (3)	0.01976 (9)	
C1	0.57846 (6)	0.3263 (3)	0.31462 (14)	0.0206 (3)	
H1A	0.5716	0.2146	0.3816	0.025*	
H1B	0.5942	0.2008	0.2588	0.025*	
C2	0.62055 (7)	0.5502 (3)	0.34383 (14)	0.0175 (3)	
C3	0.65796 (6)	0.6448 (3)	0.26657 (13)	0.0197 (3)	
H3	0.6563	0.5694	0.1938	0.024*	
C4	0.69765 (7)	0.8477 (3)	0.29468 (14)	0.0225 (3)	
H4	0.7228	0.9111	0.2411	0.027*	
C5	0.70071 (8)	0.9580 (3)	0.40082 (17)	0.0253 (4)	
H5	0.7282	1.0952	0.4204	0.030*	
C6	0.66358 (7)	0.8673 (4)	0.47815 (14)	0.0260 (3)	
H6	0.6654	0.9436	0.5508	0.031*	
C7	0.62366 (7)	0.6650 (3)	0.44994 (13)	0.0222 (3)	
H7	0.5983	0.6044	0.5035	0.027*	

### Atomic displacement parameters (Å^2^)

**Table:**

	*U*^11^	*U*^22^	*U*^33^	*U*^12^	*U*^13^	*U*^23^
Sn1	0.01130 (9)	0.01254 (9)	0.01666 (9)	0.000	−0.00051 (6)	0.000
Cl1	0.02142 (18)	0.02114 (18)	0.01692 (16)	−0.00076 (14)	0.00309 (13)	−0.00308 (13)
C1	0.0153 (7)	0.0142 (7)	0.0318 (8)	0.0000 (6)	−0.0049 (6)	0.0023 (6)
C2	0.0129 (7)	0.0138 (6)	0.0254 (8)	0.0020 (6)	−0.0044 (6)	0.0025 (6)
C3	0.0168 (7)	0.0191 (7)	0.0232 (7)	0.0026 (6)	0.0001 (6)	−0.0025 (6)
C4	0.0145 (7)	0.0216 (7)	0.0316 (8)	−0.0003 (6)	0.0022 (6)	0.0026 (6)
C5	0.0195 (8)	0.0193 (7)	0.0361 (10)	−0.0029 (6)	−0.0081 (7)	−0.0010 (7)
C6	0.0271 (8)	0.0281 (8)	0.0222 (8)	0.0000 (7)	−0.0075 (6)	−0.0023 (6)
C7	0.0204 (8)	0.0249 (8)	0.0210 (7)	−0.0011 (6)	−0.0016 (6)	0.0051 (6)

### Geometric parameters (Å, °)

**Table:**

Sn1—Cl1^i^	3.7275 (4)	C3—C4	1.388 (2)
Sn1—C1^ii^	2.143 (2)	C3—H3	0.9500
Sn1—C1	2.143 (2)	C4—C5	1.386 (3)
Sn1—Cl1	2.3695 (4)	C4—H4	0.9500
Sn1—Cl1^ii^	2.3695 (4)	C5—C6	1.384 (3)
C1—C2	1.500 (2)	C5—H5	0.9500
C1—H1A	0.9900	C6—C7	1.390 (2)
C1—H1B	0.9900	C6—H6	0.9500
C2—C3	1.394 (2)	C7—H7	0.9500
C2—C7	1.394 (2)		
			
C1^ii^—Sn1—C1	136.30 (8)	C4—C3—C2	120.80 (15)
C1^ii^—Sn1—Cl1	103.88 (4)	C4—C3—H3	119.6
C1—Sn1—Cl1	104.09 (4)	C2—C3—H3	119.6
C1^ii^—Sn1—Cl1^ii^	104.09 (4)	C5—C4—C3	120.11 (15)
C1—Sn1—Cl1^ii^	103.88 (4)	C5—C4—H4	119.9
Cl1—Sn1—Cl1^ii^	99.001 (18)	C3—C4—H4	119.9
C2—C1—Sn1	112.29 (10)	C6—C5—C4	119.69 (16)
C2—C1—H1A	109.1	C6—C5—H5	120.2
Sn1—C1—H1A	109.1	C4—C5—H5	120.2
C2—C1—H1B	109.1	C5—C6—C7	120.24 (16)
Sn1—C1—H1B	109.1	C5—C6—H6	119.9
H1A—C1—H1B	107.9	C7—C6—H6	119.9
C3—C2—C7	118.52 (15)	C6—C7—C2	120.63 (15)
C3—C2—C1	120.99 (15)	C6—C7—H7	119.7
C7—C2—C1	120.48 (15)	C2—C7—H7	119.7
			
C1^ii^—Sn1—C1—C2	177.45 (13)	C2—C3—C4—C5	0.4 (2)
Cl1—Sn1—C1—C2	−54.18 (12)	C3—C4—C5—C6	−0.8 (3)
Cl1^ii^—Sn1—C1—C2	49.01 (12)	C4—C5—C6—C7	0.5 (3)
Sn1—C1—C2—C3	−90.75 (16)	C5—C6—C7—C2	0.2 (3)
Sn1—C1—C2—C7	90.34 (15)	C3—C2—C7—C6	−0.6 (2)
C7—C2—C3—C4	0.3 (2)	C1—C2—C7—C6	178.31 (15)
C1—C2—C3—C4	−178.60 (14)		

Symmetry codes: (i) *x*, *y*−1, *z*; (ii) −*x*+1, *y*, −*z*+1/2.

## References

[bb1] Barbour, L. J. (2001). *J. Supramol. Chem.* **1**, 189–191.

[bb2] Bruker (2008). *APEX2* and *SAINT* Bruker AXS Inc., Madison, Wisconsin, USA.

[bb3] Buntine, M. A., Kosovel, F. J. & Tiekink, E. R. T. (2003). *CrystEngComm*, **5**, 331–338.

[bb4] Sheldrick, G. M. (1996). *SADABS* University of Göttingen, Germany.

[bb5] Sheldrick, G. M. (2008). *Acta Cryst.* A**64**, 112–122. 10.1107/S0108767307043930 18156677

[bb6] Shishido, K., Yoshiyuki, T. & Jiro, K. (1961). *J. Am. Chem. Soc.* **83**, 538–541.

[bb7] Westrip, S. P. (2009). *publCIF* In preparation.

